# Coordinating Metabolite Changes with Our Perception of Plant Abiotic Stress Responses: Emerging Views Revealed by Integrative—Omic Analyses

**DOI:** 10.3390/metabo3030761

**Published:** 2013-09-06

**Authors:** Jordan D. Radomiljac, James Whelan, Margaretha van der Merwe

**Affiliations:** 1 Australian Research Council Centre of Excellence in Plant Energy Biology, 4th Floor Bayliss Building M316, University of Western Australia, 35 Stirling Highway, Crawley WA 6009, Australia; E-Mails: 20170072@student.uwa.edu.au (J.D.R.); jim.whelan@uwa.edu.au (J.W.); 2 School of Plant Biology, Agriculture Building M084, University of Western Australia, 35 Stirling Highway, Crawley WA 6009, Australia; 3 Department of Botany, School of Life Science, La Trobe University, Bundoora, Victoria 3086, Australia

**Keywords:** abiotic stress, metabolic reconfiguration, metabolomics, plant, proteomics, transcriptional regulation

## Abstract

Metabolic configuration and adaptation under a range of abiotic stresses, including drought, heat, salinity, cold, and nutrient deprivation, are subjected to an intricate span of molecular pathways that work in parallel in order to enhance plant fitness and increase stress tolerance. In recent years, unprecedented advances have been made in identifying and linking different abiotic stresses, and the current challenge in plant molecular biology is deciphering how the signaling responses are integrated and transduced throughout metabolism. Metabolomics have often played a fundamental role in elucidating the distinct and overlapping biochemical changes that occur in plants. However, a far greater understanding and appreciation of the complexity in plant metabolism under specific stress conditions have become apparent when combining metabolomics with other—omic platforms. This review focuses on recent advances made in understanding the global changes occurring in plant metabolism under abiotic stress conditions using metabolite profiling as an integrated discovery platform.

## 1. Introduction

Plant growth and development are significantly affected by various stress conditions. A direct consequence of a stress perturbation is an alteration in the metabolic behavior of the cell, leading to a cascade of molecular and biochemical events that facilitate a new steady state to be reached. Depending on the development stage of the plant or type of stress applied, the phenotypical output can be associated with reduced or delayed germination efficiency [1], decreased vegetative growth [2], delayed or arrested cell cycle activity [3,4], earlier or delayed transition to flowering [5,6], decreased formation and viability of reproductive organs (for review, [7]), or accelerated senescence [2,8]. Small molecules (metabolites) play an important role during these transitions and adaptations. Perhaps the most well documented changes are the accumulation of certain metabolites, such as proline (induced upon drought, cold, osmotic, and salinity stresses, [9,10]), soluble major and minor sugars (induced upon drought, light, cold, and nutrient stresses; see also Section 3.3), glycine betaine (induced upon salt, drought, and cold stresses [11],) and amine containing compounds such as putrescine (induced upon cold and oxidative stresses, [12,13,14]), spermine (induced upon cold, oxidative, and salinity stress, [13,14]), and γ-aminobutyric acid (GABA) (induced upon salinity [15], anoxia [16], and carbon deprivation [17]). The osmotic compatibility within the cell is maintained by the accumulation of some of these metabolites (e.g. proline, glycine betaine, GABA, and sugars), decreasing the entropy levels within the cell, and allowing for folded native tertiary proteins structures to be maintained. The accumulation of other metabolites (e.g. ascorbate, glutathione, vitamin B_1_, and B_6_, [18,19,20,21]) also significantly reduces the harmful effect of reactive oxygen species (ROS) generated by abiotic stresses while ROS itself might act as an important messenger during stress responses (for review [22]). Metabolites can also serve as important allosteric regulators during abiotic stresses. Allosteric regulation involves the direct binding of a metabolite to a protein, which modifies the interaction, localization, stability, or substrate affinity of the gene product. Furthermore, the contribution of metabolites (particularly phytohormones and sugars (Section 3.3)) as important messengers during stress signal transduction under adverse growth conditions has also been well documented (for example [23]). As the metabolome (metabolite complement of a cell) represents a snapshot of the prevailing biochemical state of a particular organ or tissue under investigation, and the current estimation of the chemical complexity across the plant lineage approximates to ~200,000 chemical diverse fingerprints [24], the scope for identifying and elucidating more signaling networks upon abiotic stresses remain to be discovered. Moreover, metabolic activities respond to stress more quickly than transcriptional responses [25], suggesting that the elucidation of metabolite changes will increase our knowledge of how complex metabolic networks interact and how they are modified upon specific stresses [25]. Using metabolomics as an important diagnostic tool within the right discovery context, thus, provides a powerful means to gain novel biological insight into the metabolic regulatory network. For this purpose, this review does not attempt to detail individual metabolite changes in response to abiotic stress conditions; for a recent comprehensive review covering these aspects we refer to [25,26]. In addition, for analytical techniques associated with plant metabolite profiling, and data analyses and processing pipelines, excellent reviews have recently covered these [24,25,26,27,28]. Here, rather, we focus on the interaction between, and wealth of information that can be uncovered in studying, metabolomics combined with other molecular platforms during abiotic stress responses. The overall result is a more thorough and intricate understanding of the global adaptation patterns and hierarchical regulation structures that prevail during abiotic stress conditions. 

## 2. Transcriptional Regulation of Metabolic Networks

Key to our understanding of the central dogma of molecular biology is the linear progression of molecular events associated with it; initiated with the encoding and replication of the genomic information contained in the DNA of an organism. This gives rise to the production of messenger RNA (mRNA) that will allow for differential gene expression patterns to emerge during the process of transcription. The mRNAs are subsequently processed by an array of splicing and editing mechanisms before exiting the nucleus to the cytoplasm. Within the cytoplasm, the mRNAs will interact with large ribosomal complexes which will translate the information contained on the mRNA molecules, leading to protein synthesis. Each protein synthesized can then catalyze a set of biochemical reactions leading to the dissipation of a given substrate (metabolite x) to its biochemical (end) product (metabolite y). Until recently, this widely perceived interpretation of a metabolic pathway was commonly applied (and perhaps naively the only considerable viewpoint) when considering and interpreting the biological meaning of transcriptomic, proteomic, or metabolomic datasets when viewed in isolation. The pitfalls associated with this assumption have especially become apparent when combining two or more –omic technologies in order to obtain a more holistic view of metabolism. For a number of examples metabolic changes do follow the conventional transcriptional response. For instance, characterization of the core aerobic and anaerobic responses in rice have identified that decreased transcript abundances in sucrose catabolism or tricarboxylic acid (TCA) cycle reactions corresponds to decreased hexose equivalents and organic acids, respectively [29]. Consequently, a metabolic switch to fermentative metabolism is apparent.

However, what happens if such a clear progression (correlation between transcript (or protein) and metabolite levels) is not eminent in the dataset? How do metabolomics (and associated bioinformatic tools) aid in resolving such (apparent) discordances, and how will this influence how we evaluate metabolite data in future? Perhaps, the first important aspect is that the unequivocal identification and robustness in the dataset(s) have to establish. Currently, metabolite profiling efforts primarily focus on hyphenated chromatography based technology coupled to mass spectrometry (gas chromatography mass spectrometry (GC MS) or liquid chromatography mass spectrometry (LC MS)) high throughput techniques to profile highly to moderately abundant metabolites. In order to achieve this, unequivocal metabolite identification relies on the establishment and verification of custom metabolite libraries that rely on retention time correction indexes (retention index, RI) and matching mass-to-charge *(m/z)* identifiers. While some search algorithms are using more quantitative means of matching spectra to specific metabolites, the overall generalization still prevail to match 4–6 of the most unique *m/z* identifiers to a specific metabolite within a specific RI window. However, metabolites that share similar *m/z* identifiers, and elute in close proximity will either be misidentified using this methodology or would not be distinguished from metabolites sharing similar chemical properties. Improvements in recent MS technologies have led to the development of mass accuracy instrumentation, which significantly enhance the elucidation of metabolite identities that share similar spectra information. Moreover, technologies such as nuclear magnetic resonance (NMR) provide the most unambiguous information regarding the structural properties of a small molecule. While the use of this instrumentation has several limited capacities in terms of the sensitivity of detection, new applications such as coupling NMR and hyphenated technologies will significantly advance the profiling and identification of distinct metabolites, and allow for more robust metabolite data reporting. Improved peak resolution by using two-dimensional (or multi-dimensional) GCxGC time of flight mass spectrometry (TOF MS) has also been achieved to significantly improve separating power, and allow for a higher resolution capacity [30]. Several artifacts may also arise from derivatization steps during sample preparation of nonvolatile metabolites for GC MS analyses [31]. As an example, the conversion of arginine to the trimethylsilyl (TMS) derivative of ornithine has been previously documented [32], while the systematic detection and documentation of artifacts arising particularly from GC MS based experiments have been collated into in house databases (e.g. [33]). One approach to address such artifact formation is through the classical addition of authentic standards or spiking experiments. The annotation and quantification of metabolites can also be improved by different stable isotope labeling strategies that, in combination with standardized database searches and elemental mass calculations, allows for reduced chemical background noise and increased confidence in the elemental formula annotation [34]. A highly sensitive, ultrafast (15s) isotope-derivatization reagent, d_0_-/d_6_-2, 4-dimethoxy-6-piperazin-1-yl pyrimidine (DMPP), has also been developed to specifically derivatize carboxylic analytes, which will reduce chemical noise [35], leading to a greater confidence in the unequivocal identification of these acidic compounds. Computational annotation methods to assist in refining metabolite annotations have also been developed (e.g. [36]), and will continue to improve the accuracy that metabolites are identified with.

Apart from metabolite identification, any other apparent discrepancy in metabolic changes also has to be rigorously evaluated in the biosynthetic context. Customized in-house and publicly available software packages have been developed for standalone metabolomic data and pathway enrichment analyses that greatly aid in statistically assessing whether metabolite changes corresponds to whole pathway enrichment analyses (e.g. [37,38,39]) or graph-clustering algorithms in order to understand the organization of metabolic functional modules [40]. While such analyses have not been strictly applied to plant specific abiotic stress metabolite profiles to date, the integration of these applications greatly accelerates and creates confidence in the information generated from metabolite profiles to determine the metabolic pathway activity associated with the particular perturbation. Other bioinformatic tools to discriminate and interrogate metabolite data include both supervised and unsupervised visualization techniques, including batch learning self-organizing map analysis (BL SOM) [41], orthogonal projection to latent structures discriminant analysis (OPLS DA), principal component analysis (PCA) and Bayesian independent component analysis (ICA) in order to reduce the dimensionality or non-overlapping information in the data [42]. In addition, in a complementary approach, metabolomics and other—omic technologies could be performed in parallel and, for a combined metabolomics and transcriptomic approach, the output can be visualized; overrepresentation and other interrogative statistical analyses performed [43,44,45,46]. A number of studies have successfully integrated data from metabolomics to transcriptomics, and even enzyme activities [47,48,49,50], attesting to the value in using parallel approaches to understand plant metabolism (see also below). This review highlights several of these combined approaches in order to understand specific abiotic stresses (or abiotic stress responses in general), and the advancement of knowledge made in understanding the molecular context of metabolite changes through both targeted and untargeted profiling techniques. These metabolic changes are specifically discussed for their alternative roles in metabolism, ranging from structural modifiers of nucleic acids to important mediators or indicators of signal transduction mechanisms, concepts largely ignored when interpreting the context of metabolic changes during stress perturbations.

## 3. Metabolic Regulation of Transcriptional Networks

Any disagreement between transcriptomic and other –omic (proteomic and metabolomic) datasets are usually interpreted as an additional layer of regulation either occurring on a post-transcriptional or—translational level (although the direct comparison between technological platforms and sensitivity and analytical capacity of instrumentation should also be taken into account) [51]. The intriguing observation that certain metabolites can exert a transcriptional response preceding the induction or independent of its own biosynthetic gene expression has, however, emerged in the last years [49,52]. In addition, the exogenous application of certain metabolites that modulate or mimic abiotic stress responses [52,53] also attribute to the complexity of interpreting metabolic changes in its strict pathway context. The direct influence of prevailing metabolite levels on gene and protein expression or activity is usually referred to as metabolic regulation, ranging from simple allosteric regulation to the emergence of direct interaction of metabolites with nuclear gene expression (NGE) during retrograde signaling (Section 3.2). Regulation strategies are also most likely unidirectional as transcriptional regulation of metabolic changes will lead to the metabolic induction of transcriptional signal transduction pathways. In addition, overlapping abiotic stresses that act either synergistic or antagonistic in behavior might exhibit phases of both. As an example, the cold acclimation response and subsequent interaction with carbon availability exhibit both transcriptional and metabolic regulation strategies [54]. Thus, metabolites can exert its effect(s) on multiple hierarchical levels of control, displaying much more versatile roles than previously anticipated.

### 3.1. Metabolic DNA and RNA Structural Modifiers

Metabolites are not usually considered to have a direct interaction with genomic information (apart from forming the backbone and building blocks of this macromolecule); however, molecular evidence suggests a direct metabolite mediated modulation of chromatin structures. In plants, the monophosphorylated isomer of the phospholipid phosphatidylinositol (PtIns), phosphatidylinositol 5-phosphate (PtIns5P), interacts and binds directly to the PHD domain of *Arabidopsis* trithorax like factor, ATX1 [55]. ATX1 is an epigenetic factor that modulates chromatin structures by methylation of the N-terminal lysine 4 (K4) of histone H3 (H3Kme3) [56] but acquire target specificity as only specific genes such as the transcription factor, *WRKY70* [56], or the flowering repressor gene, *FLOWERING LOCUS C* [57], are transcriptionally activated by ATX1. In response to abiotic stresses (in particular salt, osmotic, and drought stress [55,58]), PtIns5P levels increase significantly, leading to the direct binding of cytosolic PtIns5P to ATX1. This interaction prevents the nuclear import of ATX1, leading to decreased *WRKY70* expression [59]. WRKY70 is implicated as a central convergence node between jasmonic and salicylic acid mediated signaling networks [60], and negative regulator of leaf senescence [61]. Such metabolite mediated control over epigenetic chromatin remodeling potentially has far reaching consequences, as it can be directly linked to phenotypical outputs associated with senescence and flowering aberrations during stress conditions. Moreover, small molecule modulation of chromatin structure and its subsequent effects can be experimentally employed to distinguish and clarify between the contentious subject of a transient DNA methylation state (influencing seed filling and subsequent germination efficiency) and trans generational heritable epialleles (linked to an increase in recombination frequency [62]), associated with the “memory” or “priming” effect that is frequently observed in the offspring of parents that are exposed to abiotic stresses.

Another unexpected interaction for small molecules in plant metabolism involves the existence of metabolite binding riboswitches. Riboswitches are mRNA elements that control gene regulation or resulting protein translation via a conformational change in the RNA structure upon direct binding of small interacting molecules. In bacteria and archaea, more than a hundred putative metabolite/small RNA (siRNA) responsive RNA sensors are predicted to exist [63], with 13 experimentally validated elements that bind metabolites specifically (namely purines (adenine, guanine), S-adenosyl-homocysteine (SAH), S-adenosyl methionine (SAM), tetrahydrofolate, pre-queuosone_1_, lysine, glycine, glutamine, glucosamine-6-phosphate, flavin mononucleotide (FMN), cyclic di-GMP, adenosyl-cobalamin/vitamin B_12_, and thiamin pyrophosphate (TPP)). In plants and fungi, however, only the TPP riboswitch has been identified to date, with the other known riboswitches not conserved in the plant lineage [64,65]. The plant riboswitch is located at the 3' untranslated region (UTR) of the thiamin monophosphate (TMP) biosynthetic gene, *THIAMIN C SYNTHASE* (*THIC*). In *Arabidopsis*, two other enzymes, namely TH1 and THI1 are also involved in TMP biosynthesis; however, in contrast to cytosolic localized THIC, occur in the chloroplast. TMP is subsequently dephosphorylated into thiamin, which is then pyrophosphorylated into TPP by cytosolic thiamin pyrophosphokinases (TPKs), TPK1, and TPK2 ([66], and references therein). In the nucleus, direct TPP binding to the riboswitch leads to the intron splicing of the catalytic domain of *THIC* for possible nonsense mediated decay, leaving only the riboswitch structure and thereby controlling endogenous thiamin and TPP levels [66]. The mechanism of plant TPP riboswitch control over metabolism has not been shown to have a direct role in abiotic stress sensitivity. However, exogenous application of thiamin and TPP confer enhance stress tolerance to high light, cold, osmotic, salinity and oxidative stress conditions [19,67]. It is also able to rescue the ROS sensitive *ascorbate peroxidase1* mutant from oxidative stress by decreasing protein carbonylation events and H_2_O_2_ production [19]. Abiotic stresses increase the endogenous levels of TMP, thiamin and TPP as a direct consequence of the induction of its own biosynthetic gene (*THIC, THI1, THI1,* and *TPK1*) expression [19]. Furthermore, TPP is an essential cofactor for the tricarboxylic acid (TCA) cycle enzyme complexes, pyruvate dehydrogenase (PDH) and 2-oxoglutarate dehydrogenase (2-OGDH), and for the α-ketose transketolase (TK) homodimer of the pentose phosphate pathway. Consequently, TPP availability controls the rate of carbohydrate oxidation through these pathways [66], both pathways (as well as carbon statuses, see Section 3.3) significantly affected by abiotic stresses. While *THIC* promoter activity is controlled by the circadian clock [66], the expression of *THI* and *THI1* are dependent on the stress inducible phytohormone, abscisic acid (ABA) [67]. In contrast, *TPK* expression shows no variation in response to abiotic stresses, ABA or circadian regulation [68]. In light of these observations, the dynamic relationship between the subcellular thiamin and TPP biosynthetic pathways and subsequent involvement of the TPP riboswitch (and the mutated riboswitch variant [66]) upon abiotic stress conditions would provide an interesting opportunity to explore the mechanistic basis for thiamin metabolism upon (particularly) oxidative stresses. The prospect of also engineering plants with customized riboswitches to transiently respond to changes in metabolite levels is a novel avenue to explore in future for enhancing abiotic stress tolerance without growth penalties or aberrant phenotypic variations usually observed for constitutive gene alterations grown under non stressed growth conditions.

### 3.2. Retrograde Signaling

Communication between organelles and nucleus is imperative to ensure a coordinated orchestration of avoidance or tolerance mechanisms in response to various stress stimuli. Plant organelles produce multiple retrograde signals to alter and coordinate nuclear gene expression (NGE) with organellar gene expression in order to optimize and activate defense, avoidance, or tolerance mechanisms during adverse growth conditions [69,70,71]. In plastids, certain metabolites, including tetrapyrrole Mg-protoporphyrin (Mg-Proto IX, induced during high light stress, [72]), 3'-phosphoadenosine 5'-phosphate (PAP, induced during drought or high light stress, [73]), methylerythritol cyclodiphosphate (MecPP, induced during wounding or high light stress, [74]), ROS (induced during oxidative or high light stress, [75]), ABA, or β-cyclocitral (induced during oxidative or high light stress, [76]) have all been reported to accumulate in response to various abiotic stresses and implicated as important signals during organellar retrograde signaling.

The best documented plastid retrograde system is that involving intermediates of pigment synthesis identified using a forward genetic screen, identifying *genome uncoupled* (*gun*) mutants [72] in which the expression of light harvesting chlorophyll a/b binding (*LHCB*) is uncoupled from the functional state of chloroplasts. Five of the six *gun* mutants, *gun2-6*, have impaired flux through the tetrapyrrole biosynthesis pathway, accumulating lower levels of Mg-Proto IX, an intermediate in chlorophyll biosynthesis [72]. Whilst initial reports established a direct correlation between Mg-Proto IX levels and transcript abundances, subsequent studies with more sensitive technologies have revealed that Mg-Proto IX transiently accumulate [77] while mutants or chemical inhibition of the chlorophyll biosynthesis pathway lack a clear correlation between nuclear gene expression and the tetrapyrrole intermediate [78,79]. Studies with the gain of function *gun 6-1D* mutant has further implicated heme levels, rather than Mg-Proto IX, as the most promising candidate to coordinate photosynthesis associated nuclear gene (PhANG) expression with chloroplast development [80]. Thus, at present it is not possible to distinguish between whether a direct interaction of Mg-Proto IX or heme activates transcriptional NGE, and whether Mg-Proto IX/heme only form intermediate molecules in the retrograde signal transduction pathways. In contrast, 3'-phosphoadenosine 5'-phosphate (PAP) levels accumulate in response to drought and high light stresses and have been shown to alter RNA metabolism by directly inhibiting 5' to 3' exoribonuclease activity, leading to increased NGE [73]. The isoprenoid precursor MEcPP has also been identified as a retrograde signaling molecule through identification of a genetic screen designed to identify genes involved in the regulation of *HYDROXYPEROXIDE LYASE* (*HPL*), a nuclear stress inducible gene that encodes a plastidial localized protein leading to adaptation responses upon biotic stress conditions [74]. The resulting *ceh1* mutant encodes for 1-hydroxy-2-methyl-2-(E)-butenyl-4-diphosphate synthase (HDS), responsible for catalyzing the conversion of MEcPP to hydroxymethylbutenyl diphosphate (HMBPP) in the plastidial methylerythitol phosphate (MEP) pathway. In contrast to the *gun* mutants, *ceh1* does not coordinate PhANGs expression. Rather, the accumulation of MecPP increase salicylic acid (SA) levels through modulation of the SA biosynthetic *ICS1* transcript abundance and lead to *Pseudomonas* resistance [74].

Thus, while a number of putative metabolic mediators of plastidial retrograde signaling have been identified to date (based either directly on metabolite accumulation patterns or through genetic screens identifying biosynthetic components), technical advances and approaches to study and elucidate the precise molecular mechanisms are still largely limited [71]. Furthermore, multiple independent signaling pathways might operate in parallel, or are only switched on during specific perturbations which increases the complexity associated with understanding and integrating organellar crosstalk. Thus, while the identification of metabolic changes is possibly the first steps towards elucidating certain chemical or molecular signaling pathways that might be involved in retrograde signaling, more rigorous analytical approaches and subcellular advances [71] will provide the validation and context of metabolic changes associated with organellar dysfunction.

### 3.3. Sugar Signaling

Not all metabolites have a direct influence on the molecular network; alterations in the endogenous metabolite levels could also reflect a change in the molecular function of its gene products. Clearly, the best and most extensively studied example of this is the sugar signaling network. Sugars participate in glycolysis as the main respiratory carbon source to fuel the energy supply of the cell. The first committed step towards mitochondrial respiration involves the phosphorylation of glucose and fructose to their respective hexose phosphorylated C6 intermediates via hexokinase 1 (HXK1) or isomerization of the aldose or ketose phosphorylated C1 derivatives to their respective hexose-6-phosphate moieties. HXK1 is a conserved orthologous protein of the outer mitochondrial membrane [81,82] linking cytosolic glycolysis to mitochondrial metabolism [83,84]. A loss of function screen identifying *glucose-insensitive* (*gin*) mutants has elucidated HXK1 as an important regulatory node in sensing internal glucose levels [85]. In this regard, HXK1 localizes to the nucleus and activates nuclear gene expression through protein complex formation with the vacuolar H^+^ ATPase B1 (VHA-B1) and 19S regulatory particle of the proteasome subunit (RPT5B) [85]. The biosynthetic activity of HXK1 can be uncoupled from its sensing role, as catalytic inactive variants can complement the glucose sensitive phenotypes, including reduced shoot and root growth, delayed flowering and senescence, and altered sensitivities to the growth hormones auxin and cytokinin [86]. However, low glucose levels can also mediate sugar signaling pathways independent of HXK1 signaling [87] (see also below). During a range of abiotic stresses (see Table 1 for specific stresses), endogenous sugar levels change significantly across a range of stress stimuli (Table 1).

**Table 1:** Environmental perturbations lead to alterations in soluble sugar levels, gene expression patterns and enzyme activities in *Arabidopsis thaliana*.

Abiotic stress	Soluble sugar	Sugar response	Gene expression	Gene	Gene model	Enzyme activity	Enzyme	Reference
Cold	gluc, fruc	increase	increase	*FXK3*	At5g51830	nd	nd	[88]
				*GOLS3*	At1g09350			
				*HXK2*	At2g19860			
				*RS5*	At5g40390			
				*SPS1*	At5g11110			
				*SPS1F*	At5g20280			
			decrease	*SSR*	At1g79440			
Nitrogen (N) deficiency	∑sugar (suc + gluc + fruc)	increase	increase	*TPP B*	At1g78090	increase	GlucoK	[89] [90]
				*PGM*	At1g78050		FUM	
				*A/N-Inv D*	At1g22650			
				*G6PDH*	At1g24280			
				*PGDH*	At1g64190			
				*ABI4*	At2g40220			
			decrease	*TPS3*	At1g17000	decrease	AGPase	
				*CWINV4*	At2g36190		NADP-GAPDH	
							NADP-IDH	
							NR	
							GS	
							AspAT	
Potassium (K) deficiency	suc, gluc, fruc	increase	−	nd	nd	increase	FrucK	[91]
							NADP-ME	
							GS	
							GOGAT	
						decrease	NR	
Phosphate (P) deficiency	suc, gluc, fruc	increase	increase	*PPCK1*	At1g08650	increase	cFBPase	[90]
				*PPCK2*	At3g04530		SPS	
				*BAM5*	At4g15210		PEPCase	
				*GWD3*	At4g24450		GS	
				*GBS1*	At1g32900		GDH	
				*GPT2*	At1g61800		ShiDH	
				*ADG2*	At5g19220			
				*APL3*	At4g39210			
				*SPS4*	At4g10120			
				*SPP1*	At1g51420			
				*SUS3*	At3g43190			
			decrease	*FLN1*	At3g54090	decrease	PFP	
				*FLN2*	At1g69200			
Carbon availability	gluc	Increase (exogenous feeding)	increase	*ABI4*	At2g40220	nd	nd	[92]
				*ABI5*	At2g36270			
				*CTR1*	At5g03730			
Combined heat and drought	suc, tre, gluc, fruc	increase	decrease	*AMY1*	At4g25000	nd	nd	[93]
				*BAM5*	At4g15210			
				*C/VIF1*	At1g47960			
				*FLN1*	At3g54090			
				*G6PDH6*	At5g40760			
				*HXK2*	At2g19860			
				*PSL5*	At5g63840			
				*SPS1F*	At5g20830			
				*SPS1*	At5g20280			
Heat	suc, gluc, fruc	increase	increase	*NDB1*	At4g28220	nd	nd	[93]
				*VHA-A*	At1g78900			
				*GOLS1*	At2g47180			
				*A/N-INVC*	At3g06500			
Drought	suc, gluc, fruc	increase	increase	*APL3*	At4g39210	nd	nd	[93]
				*SPS1*	At5g11110			
				*SUS1*	At5g20830			
				*G6PDH6*	At5g40760			
Drought	suc	increase	increase	*CWINV1*	At3g13790	nd	nd	[94,95]
				*CINV1*	At1g35580			
				*GDH1*	At5g18170			
				*GDH2*	At5g07440			
				*VAC-INV*	At1g12240			
			decrease	*ADK1*	At3g09820			
				*GDH3*	At3g03910			
Anoxia	gluc, fruc	increase	increase	*ADH1*	At1g77120	nd	nd	[16]
				*SUS4*	At3g43190			
Salinity	suc, gluc	increase	increase	*PYD4*	At3g08860	nd	nd	[96]
				*BAM1*	At4g15210			
				*GPAT5*	At3g11430			
			decrease	*PDF1.2b*	At2g26020			
				*PDF1.2*	At5g44420			

Abbreviations: ABI; ABA insensitive, ADG; ADP glucose pyrophosphorylase, ADH; alcohol dehydrogenase, AMY; alpha-amylase, A/N-INVC; alkaline/neutral invertase C, APL; ADP-glucose pyrophosphorylase, BAM; beta-amylase, cFBPase; cytosolic fructose bisphosphatase, C/VIF; cell wall/vacuolar inhibitor of fructosidase, CWINV; cell wall invertase, CINV; cytosolic invertase, SSR; succinic semialdehyde reductase, fruc; fructose, FrucK; fructokinase, FXK; fructokinase, FLN; fructokinase-like, FUM; fumarase, gluc; glucose, GBS; granule-bound starch synthase, GlucoK; gluconokinase, GOGAT; ferredoxin-glutamine-2-oxoglutarate aminotransferase, GOLS; galactinol synthase, GPT; glucose 6 phosphate/phosphate transporter, GS; glutamine synthetase, GWD; glucan-water dikinase; G6PDH; glucose-6-phosphate dehydrogenase, GDH; glutamate dehydrogenase, GOLS; galactinol synthase, GPAT; glycerol-3-phosphate sn-2-acyltransferase, HXK; hexokinase, NADP-ME; NADP-dependent malic enzyme, nd; not determined, NDB1; NADH dehydrogenase B1, NR; nitrate reductase, PDF1.2; plant defensin 1.2, PDF1.2b; plant defensin 1.2B, PEPCase; phosphoenolpyruvate carboxylase, PFP; pyrophosphate-fructose-6-phosphate phosphotransferase, PGDH; 6-phosphogluconate dehydrogenase, PGM; phosphoglucomutase, PPCK; phosphoenolpyruvate carboxylase kinase, PSL5; priority in sweet life 5, PYD4; pyrimidine 4, RS5; raffinose synthase 5, ShiDH; shikimate dehydrogenase, SPP; sucrose phosphate phosphatase, SPS; sucrose phosphate synthase, SPS1F; sucrose phosphate synthase 1F, suc; sucrose, SUS; sucrose synthase, tre; trehalose, TPP; trehalose-6-phosphate phosphatase, TPS; threhalose-6-phosphate synthase, VAC-INV; vacuolar acid invertase, VHA-A; vacuolar ATP synthase subunit A.

Prevailing sucrose levels are sensed by the sucrose non fermentation1 related protein kinase 1 (SnRK1), a conserved heterotrimeric protein kinase protein complex consisting of KIN10 and KIN11 subunits. KIN10 is the orthologue of the mammalian AMP activated protein kinase (AMPK), while KIN11 is the orthologue of the yeast sucrose non fermenting (SNF1) protein kinase, activated in response to low cellular glucose levels [97]. Plant SnRK1s modulate sugar and ABA signaling pathways in response to energy depleting perturbations by controlling stress responsive gene expression [98]. Thus, while KIN10 and KIN11 are predominantly localized in the cytoplasm, upon carbon and energy limitation, and form a protein complex that is sequestered to the nucleus where it has key transcriptional regulating capabilities. SnRK1 transcriptional activity is inhibited by the levels of the endogenous phosphorylated sugars, trehalose-6-phosphate (T6P) and glucose 6-phosphate (G6P) [99,100,101], and upon phosphate starvation [102]. The phosphorylated sugar, T6P, also promotes thioredoxin mediated redox activation of ADP-glucose pyrophosphorylase (AGPase) involved in starch biosynthesis in response to cytosolic sugar levels [103]. Overexpression of the basic leucine zipper domain transcription factor, bZIP11 further leads to the accumulation of endogenous T6P levels [104]. However, sucrose also represses the translation of bZIP11 leading to sucrose regulated changes in amino acid metabolism [105]. Multiple lines of evidence now exist for the direct correlation between T6P and sucrose levels (e.g. [105,106]), and this association has been greatly accelerated by the accurate identification and quantification of the metabolites in question. T6P also acts as a shoot apical meristem (SAM) localized sugar signal for regulating the onset of flowering in response to environmental perturbations, including day length, temperature, hormonal cues, and carbohydrate availability [106]. KIN10 overexpression further exhibit glucose hypersensitivity (see also below) and alters carbon and nitrogen metabolism by controlling the expression of certain genes such as nitrate reductase (NR) and AGPase [98]. Upon hypoxic conditions, heterologous KIN10 overexpression leads to increased longevity of rice and *Arabidopsis* under submergence due to increased expression of alcohol dehydrogenase (*ADH1)* and pyruvate decarboxylase (*PDC1)* [107]. The expression of these genes can be restored by sucrose addition [107].

Interestingly, KIN10 also interacts with cyclin dependent kinase E; 1 (CDKE;1), a regulator identified in the mitochondrial ALTERNATIVE OXIDASE (AOX) dependent retrograde signaling pathway and part of a nuclear transcriptional mediator complex [108]. Orthologues of both KIN10 and other subunits of the mediator complex have also been identified in *Neurospor*a *crassa* to regulate *AOX* expression [109], suggesting an evolutionary conserved response in crosstalk between components of retrograde and sugar signaling (see also below). While the exact molecular mechanism is not clear, *cdke;1* mutants display increased sugar levels upon unstressed growth conditions, while *aox1a* and *kin10* mutants have reduced sugar levels compared to their respective controls during the photosynthetically active period (Radomiljac and Whelan, *unpublished results*). The *aox1a, kin10* and *cdke;1* mutants all show normal developmental phenotypes grown under non stressed conditions, suggesting that the signal cascade operating between sugar signaling, retrograde signaling, and phenotypical alterations upon abiotic stresses can be elucidated in reverse genetic studies combined with phenomic and metabolomics approaches. While this has not been addressed to date, it will be an exciting prospect in future in order to use metabolomics directed approaches to understand mitochondrial retrograde signaling and metabolic activities to enhance plant fitness and survival during limiting growth conditions. Furthermore, abscisic acid insensitive 4 (ABI4) has also been identified as a central regulator of both mitochondrial AOX dependent [110] and chloroplast dependent [111] retrograde signaling, modulating AOX1a responses during high light and drought stress [112]. An extensive crosstalk between ABI4 and sugar signaling has been demonstrated [92], while *abi4* mutants display increased salt tolerance [113]. Thus, while organellar retrograde signaling share a significant degree of crosstalk [114], metabolic changes related to sugar metabolism, signaling, and responsive gene expression further implicates a higher degree and extent of intracellular small molecule communication during abiotic stresses. In addition to the influence of sucrose and glucose mediated signaling, fructose levels can also be sensed *in planta*. Screening several enzymes in fructose metabolism by luciferase reporter screens in transient *Arabidopsis* mesophyll protoplasts have identified *fructose insensitive* (*fin*) mutants based on their inability to affect photosynthetic gene expression [115]. *fin1* encodes for the metabolic enzyme fructose-1,6-biphosphatase (FBPase) [115], responsible for fructose-1,6-biphosphate steady state levels. Inhibition of cytosolic and plastidial FBPase activities severely limit photosynthesis in potato plants [116,117]. In addition, plastidial localized fructokinase-like (FLN) proteins are targets for a plastidial thioredoxin, TRX *z*, that regulate plastid-encoded RNA polymerase (PEP) dependent transcription in plastids [118], suggesting that subcellular sugar metabolism and/or signaling have profound effects on coordinated nuclear organellar responses.

TARGET OF RAPAMYCIN (TOR) proteins are evolutionarily conserved Ser/Thr kinases that control growth signaling pathways in response to stress signals through modulation of ribosome biogenesis, translation and primary metabolism [119]. Upon osmotic stress, TOR interacts with RAPTOR1 (a regulatory protein of TOR) to regulate the activity of S6 kinase, a protein conferring osmotic hypersensitivity to transgenic *Arabidopsis* plant [120]. TOR also interacts with LST8 proteins that modulate plant growth, flowering, nitrate assimilation and sugar metabolism [121]. Although, the most recent player to be involved in integrating cellular sugar and energy depleting statuses, TOR signaling, in combination with SnRK1 signaling, has been implicated as an important regulatory component during mitochondrial retrograde signaling. As kinases, both SnRK1 and TOR proteins are involved in the post-translational phosphorylation modification of other target proteins. The alteration in the phosphorylation status due to perturbations in mitochondrial membrane potential has also been proposed to be a mechanism for the translocation of putative transcription factors encountered on the outer mitochondrial membrane [81,82] to the nucleus [122]. In addition to the identification of the kinase CDKE;1 as a regulator of *AOX1a* expression (but exhibiting post-transcriptional regulation of AOX1a protein abundance) [108], an enigmatic question is the identification of the main metabolic signals that activate kinases and transduce the phosphorylation status. Furthermore, the overlap between sugar signal kinases and the retrograde kinases are a striking convergence point that needs to be explored in future.

Thus, while sugar accumulation could potentially increase the osmotic potential within the cell (see introduction) or fuel respiration through glycolysis, the TCA cycle and mitochondrial oxidative phosphorylation, a third fundamental consequence is the participation of sugars in specific signal transduction mechanisms during abiotic stress conditions. The specificity of the sugar signaling response (i.e. glucose, fructose and sucrose signaling can be distinguished from each other) attests to a functional role for the individual sugars apart from an osmoprotectant or respiratory intermediate. In regards to the crosstalk and mechanisms operating between the different sugars, and the interactions within the rest of the cell; these are exciting research avenues where metabolomics driven endeavors can greatly provide novel insight into the molecular mechanisms underlying the specific abiotic stress responses. A key question arising from the data presented in Table 1 (and in context with this review) is the establishment of whether the same core sugar signaling pathways are activated and/or transduced during the different abiotic stresses. Despite the individual sugars showing a striking similarity in response to a wide range of stresses, with multiple marker genes correlating with these (Table 1), addressing the robustness in the response of sugar signal perturbations (and the upstream cascade of events) is still largely lacking. The parallel time course analyses of the perception and initiation of sugar signaling, as well as the molecular players activated upon specific abiotic stresses, will help elucidate some of the key components of this signaling response.

One other aspect to address during the evaluation of metabolite changes is the robustness or diversity in metabolic activities and steady state metabolite abundances on species and developmental level. While the aforementioned discussion has exclusively focused on the molecular pathways or mechanisms associated with metabolite linked changes in the model species, *Arabidopsis thaliana*, a great deal of literature has also accumulated in other important crop and forage species where expansive metabolomic experiments have been performed upon various specific abiotic stresses (for example, salinity and drought stress [123,124,125,126,127]). This data could help to evaluate the translatability of metabolic changes observed, and more importantly, the associated molecular mechanism(s) underlying the response, from model to important crop species. As an example, combined ionomics, transcriptomic and metabolomics studies on *Lotus* genotypes illustrated that the identification of genotype-specific molecular responses may provide a more realistic rational for the design and implementation of tolerance traits under salt stress. The differential display of osmolyte and other small molecule accumulation upon drought stress of different *Lotus* genotypes suggest that coordinating metabolic activities are orchestrated in a finely tuned manner [125,126], and might need more global approaches to understand these adaptive mechanisms (see also below). Similarly, investigating metabolite changes in different maize hybrids in greenhouse grown conditions do not correlate with water deficit tolerance traits observed under field conditions [128], suggesting that finding distinct metabolic cues and underlying activities require molecular biology and agronomical approaches to converge in order to find practical field based solutions to improve plant growth and performance upon non optimal growth conditions.

## 4. Integration with the Other—Omics Platforms: Phenomics, Genomics, Proteomics, and Metabolomics

While this review has largely focused on transcript-metabolite cohort changes, and the signal transduction pathways they participate in, metabolomics can be applied to a diverse range of –omic platforms to gain functional insight into physiological and molecular responses during abiotic stresses. The application of metabolomics to phenomics, genome wide association (GWA) and recombinant mapped populations, genome and quantitative trait loci (QTL) sequencing efforts have provided the timely opportunity to explore the molecular and biochemical basis and pathway interactions for complex plant abiotic stress interactions [48,129]. As an example, in a GWA mapping strategy applied to 289 maize inbred lines, the biochemical composition of 26 metabolites were strongly associated with single nucleotide polymorphisms (SNPs), including 15 distinct SNP-metabolite correlations that underpinned the genetic variance, observed in these hybrids [130]. Such discovery modules will greatly accelerate the future directives to understand the complexity associated with, and strategies to improve, plant performance upon specific abiotic stresses. For *Arabidopsis* accessions, a similar approach has been applied upon C and N limitation and, once again, suggests that the resulting metabolic networks are highly variable and specific in terms of the limiting resource [131]. Similarly, upon cold stress, ecotype variance in *Arabidopsis* accessions could identify specific lipids or lipid classes (e.g. glucose/galactosylceramide (d18:1/c24:0), monogalactosyldiacylglycerol (MGDG) 34:2, 34:3, 36:7), and triacylglyceride (TAG 52:0 and 58:9)) through ultrahigh performance liquid chromatography Fourier transform MS (UPLC FT MS) technology positively correlated with low temperature acclimation; allowing for potential metabolic biomarker discovery that can be used in breeding strategies [132].

Furthermore, while perhaps less suited to proteomic studies dealing primarily with relative protein abundances, the integration of metabolomics with quantitative proteomics and, especially, enzyme activities [48], have also yielded far greater insight into the (post-) translational metabolic regulation of plants upon, for example, limited carbon availability [48]. Metabolites can serve as transient modulators of protein function and therefore have useful applications in the dynamic signal transduction pathway that lead to the activation of abiotic stress tolerance [133]. Cross *et al.* suggests that altered biomass and growth limitations of 24 genetically diverse *Arabidopsis* accessions upon carbon deprivation is not a result of absolute metabolite levels of starch, sugars, or amino acids; but, based on the measured enzymatic capacity, related to the relative flux through the metabolic system [48]. As such, fluxomics, currently defined as the measurement of isotopic (stable or radioactive) label enrichment of metabolites (or pools) across time in organs, tissue or cell types, plays a pivotal role in linking changes in metabolite levels to that of genomic and proteomic platforms. The technologies and approaches to measure these on a more comprehensive, high throughput basis are still being developed (for recent comprehensive review, [134]). One major limitation is our largely fragmented knowledge about plant metabolism. The identification of “new” metabolites or metabolite derivatives (both in primary and specialized metabolism), as well as novel metabolic biosynthetic pathways, is imperative to interpret metabolic flux labeling and account for flux balance equations. Current flux methodologies rely thus heavily on empirical mathematical modeling to obtain a reasonable goodness of fit of obtained experimental isotopic distribution to that of curated metabolic models. As a result, fluxomics are currently more specific and applicable for tailoring experiments to resolve specific fluxes with high precision by labeling with precursor isotopes that are in close proximity to the area or flux distribution point of interest, or within a well-defined pathway. Metabolic flux can also be only truly reflective of *in vivo* conditions if each metabolite (and its resulting label enrichment) in the pathway, starting with total label uptake and utilization, can be quantitated, and these parameters need to be readily measured and be accounted for in any mass calculation equation. One way to currently gain biological insight into dynamic metabolite states is to define the degree of label enrichment within each individual metabolite (as assessed via hyphenated MS technologies), without interfering flux dynamics from it.

As an example, in *Arabidopsis* roots exposed to oxidative stress; ^13^C glucose isotopic labeling incorporation into TCA cycle organic acids, and sucrose was significantly decreased, although the absolute concentration of these respective metabolites remained unaltered [135]. Upon recovery, experiments from oxidative stress, the metabolic recovery shifts (both steady state and dynamic metabolite levels and enrichment, respectively) precedes the enzymatic activities of the majority of the biosynthetic gene products [136], suggesting that combined metabolomics/fluxomics and proteomic approaches act as important platforms that can pinpoint the metabolic step(s) involved in plant stress responses.

Another major area of advancement of metabolomic data through the combination of fluxomics, is the broader application of isotopes to plant systems grown in closed facilities supplied with different isotopic labeling precursors (^13^C, ^15^N, ^34^S) [34,137,138,139]. This approach leads to a greater confidence in current metabolite annotation and also promise to expand the plant metabolite depository needed to improve current flux balance models. Greatly aiding these assignments is improved instrumentation, including Fourier transform ion cyclotron resonance mass spectrometry (FTICR MS) and Orbitrap mass spectrometers (see also Section 2), that allows for mass accuracy and isotope elemental composition determinations. Combining such larger scale metabolite/flux studies have not been applied to plants upon abiotic stress, however, strategies to incorporate this promise to uncover unique and diverse metabolite identities and signatures that participate in various signaling and metabolic roles.

Linking chemical genomics, fluxomics and metabolite profiling technologies have the further potential to uncover novel signal transduction pathways, and also for genetic engineering strategies to transiently perturb transcript or protein levels in response to stress stimuli (reviewed in [24,140]). In addition to these experimental integration strategies, subcellular metabolomics [134,141], tissue specific metabolite profiling [142], and the spatial compartmentation of cell specific transcriptional changes upon, for example nitrogen and iron deficiency [143,144], have also provided more technical advances in refining models and hypothesis surrounding microscale metabolic changes upon nutritional stresses to date. Combined with further technologies, including fluxomics and ionomics, multidisciplinary approaches to understand abiotic stress adaptation and tolerance are rapidly expanding, and the next challenge is the systematic integration of metabolic data into its correct molecular context.

## 5. Conclusions

Metabolomics has taught us a great deal about the diversity in and dynamics of systems driven biological approaches during abiotic stresses. Ranging from osmoprotectants to allosteric regulators of protein properties, as well as direct interactors and modulators of DNA and RNA structures to alter nuclear gene expression, small molecules and the study thereof (metabolomics) have illustrated that, in combination with other –omic datasets, metabolites, proteins, and transcripts do not always “play by the rules of the game”. While it challenges us to shift our assumptions and interpretation of metabolic changes in terms of how we think metabolism operates, it also greatly attests to the complexity within biological systems. Our current understanding of data structure relationships (particularly transcript-metabolite or metabolite-metabolite comparisons), and resulting hierarchical regulation structures, have been significantly improved by addressing metabolomics driven questions by careful designing experiments that will aid in understanding the complexity and regulation mechanisms during abiotic stresses. Plant metabolomics has taught us that, while metabolomics is a powerful tool to understand specific abiotic stresses (and even how these stresses overlap), we need the integration of other platforms or genetic variability in order to understand and integrate from *what we measure* to what the relevant molecular context is of these metabolic changes. However, metabolomics as a standalone entity will still provide valuable information regarding the identification of uncharacterized metabolites and/or derivatives, greater quantification of metabolites on both tissue specific and subcellular levels, as well as the dynamic movement of soluble metabolites between cells and tissues, especially upon a range of stresses. In conclusion, metabolomics is a crucial link to decipher the molecular functional mechanisms(s) underlying specific abiotic stress responses.
